# Retrieving the chlorophyll content of individual apple trees by reducing canopy shadow impact via a 3D radiative transfer model and UAV multispectral imagery

**DOI:** 10.1016/j.plaphe.2025.100015

**Published:** 2025-03-06

**Authors:** Chengjian Zhang, Zhibo Chen, Riqiang Chen, Wenjie Zhang, Dan Zhao, Guijun Yang, Bo Xu, Haikuan Feng, Hao Yang

**Affiliations:** a School of Information Science and Technology, Beijing Forestry University, Beijing, 100083, China; b Key Laboratory of Quantitative Remote Sensing in Agriculture of Ministry of Agriculture and Rural Affairs, Information Technology Research Center, Beijing Academy of Agriculture and Forestry Sciences, Beijing, 100097, China

**Keywords:** Chlorophyll content, Shadows, Vegetation index (VI), Radiative transfer models (RTMs), Hybrid inversion model, Individual apple tree crown

## Abstract

Accurate monitoring and spatial distribution of the leaf chlorophyll content (LCC) and canopy chlorophyll content (CCC) of individual apple trees are highly important for the effective management of individual plants and the promotion of the construction of modern smart orchards. However, the estimation of LCC and CCC is affected by shadows caused by canopy structure and observation geometry. In this study, we resolved the response relationship between individual apple tree crown spectra and shadows through a three-dimensional radiative transfer model (3D RTM) and unmanned aerial vehicle (UAV) multispectral images, assessed the resistance of a series of vegetation indices (VIs) to shadows and developed a hybrid inversion model that is resistant to shadow interference. The results revealed that (1) the proportion of individual tree canopy shadows exhibited a parabolic trend with time, with a minimum occurring at noon. Correspondingly, the reflectance in the visible band decreased with increasing canopy shadow ratio and reached a maximum value at noon, whereas the pattern of change in the reflectance in the near-infrared band was opposite that in the visible band. (2) The accuracy of chlorophyll content estimation varies among different VIs at different canopy shadow ratios. The top five VIs that are most resistant to changes in canopy shadow ratios are the NDVI-RE, Cire, Cigreen, TVI, and GNDVI. (3) For the constructed 3D RTM ​+ ​GPR hybrid inversion model, only four VIs, namely, NDVI-RE, Cire, Cigreen, and TVI, need to be input to achieve the best inversion accuracy. (4) Both the LCC and the CCC of individual trees had good validation accuracy (LCC: R^2^ ​= ​0.775, RMSE ​= ​6.86 ​μg/cm^2^, nRMSE ​= ​12.24 ​%; CCC: R^2^ ​= ​0.784, RMSE ​= ​32.33 ​μg/cm^2^, and nRMSE ​= ​14.49 ​%), and their distributions at orchard scales were characterized by considerable spatial heterogeneity. This study provides ideas for investigating the response between individual tree canopy shadows and spectra and offers a new strategy for minimizing the influence of shadow effects on the accurate estimation of chlorophyll content in individual apple trees.

## 1 Introduction

The leaf chlorophyll content (LCC) and canopy chlorophyll content (CCC) are widely recognized as important indicators of a plant's physiological processes and health status[[1], [2], [3]]. The LCC is the amount of chlorophyll *a* and *b* per unit area (μg/cm^2^), and the CCC is the integrated chlorophyll content of the canopy, which can be approximated as the product of the LAI and LCC. Both provide indirect estimates of leaf/canopy N content[[4], [5], [6]]. In the context of modern smart orchards, LCC and CCC can be used as important indicators for the fine management of individual apple trees[7] and are of significant interest in clarifying individual plant growth status[8], stress levels[9,10], and photosynthetic potential[5,11]. Therefore, the accurate monitoring and spatial distribution of the LCC and CCC of individual apple trees are important for guiding agricultural production and management.

Chlorophyll has traditionally been measured using chemical assays involving sampling from each canopy and subsequent labor-intensive laboratory operations in a way that is impractical for scaling to the orchard scale [12]. Numerous leaf and canopy experiments have shown that vegetation spectroscopy is a powerful tool for assessing changes in LCC and CCC [11,13,14]. The current spectral-based approaches for chlorophyll content estimation are as follows: (1) parametric regression; (2) nonparametric regression; (3) physical model inversion; and (4) hybrid inversion [4,15].

Parametric regression relies on explicit relationships between vegetation indices (VIs) and chlorophyll content, whereas nonparametric regression requires large datasets to calibrate the models. Both approaches are extensions of data-driven methods and are generally considered to have poor spatial and temporal scalability. Physical model inversion is based on radiative transfer models (RTMs) and is performed by lookup tables or optimization algorithms. RTMs are based on well-defined physical laws and do not require measured samples for calibration, making them more suitable for spatial‒temporal generalization. This approach is commonly used for the inversion of leaf-scale chlorophyll content, but it is relatively less commonly applied at the canopy scale. The large number of input parameters required at the canopy scale leads to lower computational speed and can result in ill-posed inversion problems. The hybrid method, also referred to in recent years as knowledge-guided machine learning, combines RTMs for simulating large datasets with machine learning techniques. This approach strikes a balance between model interpretability and efficiency, making it one of the most commonly used methods for estimating canopy-scale LCC. Numerous studies have shown that RTMs ​+ ​Gaussian process regression (GPR) are more powerful than many other hybrid approaches and perform well in inversion tasks [[16], [17], [18], [19], [20]]. However, the accuracy of this method depends on the simulated dataset; thus, the challenge lies in generating sufficiently realistic and representative canopy remote sensing reflectance through simulation.

Different RTMs have been established to characterize the mechanisms of photon transport between the canopy and its surroundings. One-dimensional (1D) RTMs (e.g., PROSAIL) rely on the assumption of vegetation homogeneity, abstracting vegetation as a turbid medium, and are often used for satellite sensor data with coarse spatial resolution[21] or for homogeneous crop canopies (e.g., maize[22], wheat[2,23], and rice[24]). In regard to various individual tree covers with intricate structural elements [25], such as notable shadowing impacts and differences in branch arrangement [26], three-dimensional (3D) RTMs are clearly more appropriate, as they can address explicitly described heterogeneous canopy structures[27,28]. Many 3D RTMs with integrated canopy structures, including the discrete anisotropic radiative transfer (DART) model[29] and the large-scale remote sensing data and image simulation framework (LESS)[28], have been applied to retrieve forest or fruit tree canopy traits.

High-resolution unmanned aerial vehicle (UAV) imaging spectroscopy can accurately estimate individual canopy traits and map spatial variability because of its simultaneous multichannel acquisition and spatially explicit geographic location information [11,12,26,30]. Considerable efforts have been made to estimate structural or biochemical properties at the individual tree level, such as the ratio of photosynthetic to nonphotosynthetic portions of the canopy [26], photosynthetic capacity [11] (e.g., maximum carboxylation rate, V_cmax_, and maximum electron transport rate, J_max_), and chlorophyll or carotenoid content [12,31].

For individual apple tree canopies, the absorption and scattering properties of the leaves are easily disturbed by shadow pixels, weakening the relationship between the reflected solar radiation from the canopy and the chlorophyll content. Studies have shown that removing shadow pixels from the canopy can significantly improve the accuracy of chlorophyll content inversion [12,32]. Although high-resolution imaging from UAVs has facilitated canopy parameter inversion, it has also exacerbated the shadow effects within the canopy [33]. The adjacency effect between pixels causes some pixels to appear as artifacts (i.e., shadow pixels) in the imagery. These artifacts differ in their true reflectance, obscuring important spectral features and leading to inaccurate estimates of chlorophyll content [34]. Additionally, the complex branching structure of the canopy, the position of the sun, and the observation angle all contribute to shadow effects within the canopy [7,35].

Some scholars have attempted to elucidate the impact of shadow effects on spectral responses and parameter inversion. For satellite imagery [36], explained the relationship between the photon escape probability (fesc) and the phase angle between the satellite and the sun by utilizing a 3D RTM and multiangle satellite imagery, indirectly demonstrated that canopy shadows cause the underestimation of VIs, and then qualitatively speculated that the canopy shadow effect would lead to a decline in the inversion accuracy of downstream canopy parameters [3]. employed an inversion method using the hybrid INFORM model to quantify the impact of shadow effects on the estimation of chlorophyll content in coniferous forests at the Sentinel-2A resolution. In these low-resolution satellite images, sunlit and shadowed vegetation within a pixel are inseparable components; thus, the shadow effects within individual tree canopies are largely neglected. However, for high-resolution spectral imagery from UAVs or ground-based platforms, the shadowed pixels within the canopy become significant [33,37], especially concerning individual tree canopies. Therefore, it is essential to quantitatively determine the impacts of shadows on canopy spectra, vegetation indices, and parameter inversion for individual tree canopies before conducting extensive spatial mapping of individual tree canopies.

In studies involving the inversion of canopy chlorophyll content using high-resolution UAV imagery, shadow pixels within the canopy are typically masked using threshold methods or machine learning algorithms, yielding only sunlit pixels with high signal-to-noise ratios [[38], [39]]. However, for centimeter-resolution pixels, determining whether the shadow pixels within the canopy have been sufficiently masked by any method is challenging. Pixels containing shadows within the canopy still introduce uncertainty in the inversion of chlorophyll content. Although 3D RTMs can invert the LCC of a heterogeneous canopy, spectral response analysis of confounding factors, particularly shadows, beyond LCC is still needed. On the basis of this analysis, more robust methods for inverting the LCC should be developed. Therefore, we also aim to develop a shadow-resistant method for the inversion of chlorophyll content in individual tree canopies that does not rely on masking algorithms.

On the basis of UAV multispectral imagery and a 3D RTM, this study aims to (1) uncover the spectral response to shadows in individual apple tree crowns; (2) assess the resistance of VIs to shadows and screen VIs for the retrieving the LCC and CCC of individual tree crowns; and (3) develop a hybrid inversion model for the LCC and CCC of individual apple trees that is resistant to shadow interference and evaluate the accuracy and spatial mapping.

## 2 Materials and methods

### 2.1 Materials

#### 2.1.1 Data acquisition site

**Fig. 1:**
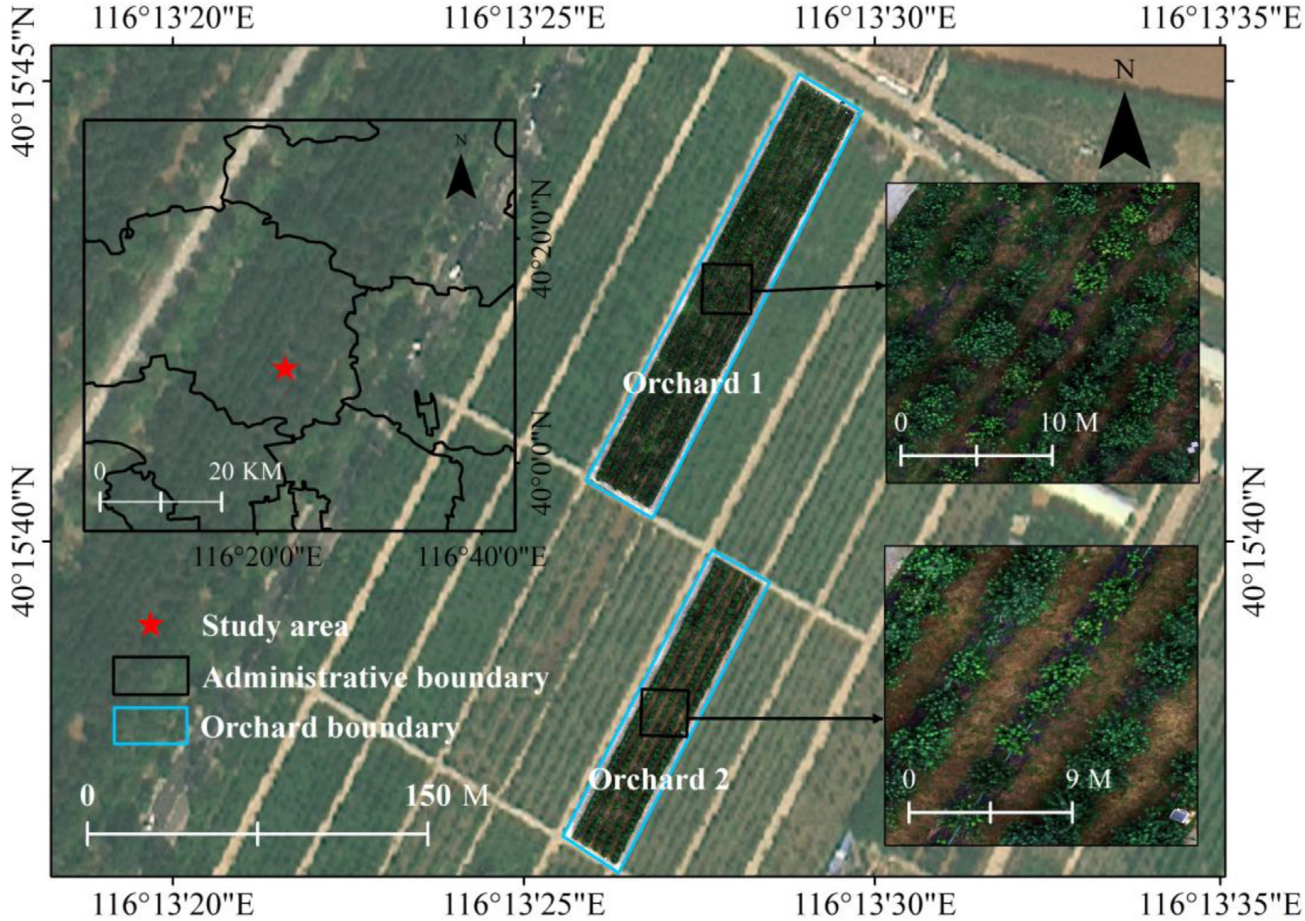
Study area and experimental orchards.

Two orchard plots planted with apple trees located in Changping District, Beijing, North China (116.133°E, 40.154°N), were chosen for this investigation (Fig. 1). The plots are located in a temperate monsoon area with a warm temperate continental monsoon climate. The average annual sunshine duration, temperature and precipitation are 2684 ​h, 11.8 ​°C and 550.3 ​mm, respectively. This climate is ideal for cultivating horticultural crops, such as apple trees.

#### 2.1.2 Acquisition of LAI data for individual apple trees

The LAI of individual apple trees was acquired on August 30, 2021, via an LAI-2200 plant canopy analyzer (PCA) (LI-COR Inc., Lincoln, NE, USA). The LAI-2200 analyzer was equipped with a fisheye lens to measure the gap fraction, and the principle of path length distribution was used to calculate the LAI. [40]. Notably, LAI-2200 measurements are sensitive to direct sunlight and therefore need to be performed when the proportion of direct light is weak to obtain the best measurement [41,42]. Prior to measuring the LAI for trees, skylight was assessed using sensors in open areas, followed by LAI measurements in four directions beneath the canopy. Each sample tree was measured three times, and the average value was recorded as the tree's LAI. Eighty-five trees were randomly chosen, and the results were compiled (Fig. S1(a)).

#### 2.1.3 Leaf chlorophyll content measurement

The chlorophyll content was determined through destructive sampling. We collected sample leaves from the field and immediately brought them to the laboratory for chlorophyll extraction. Details of the experiments and calculations can be found in our previous studies [30,32]. The statistics for Cab and CCC are shown in Fig. S1 (b), (c).

#### 2.1.4 Measurement of optical properties

**Fig. 2:**
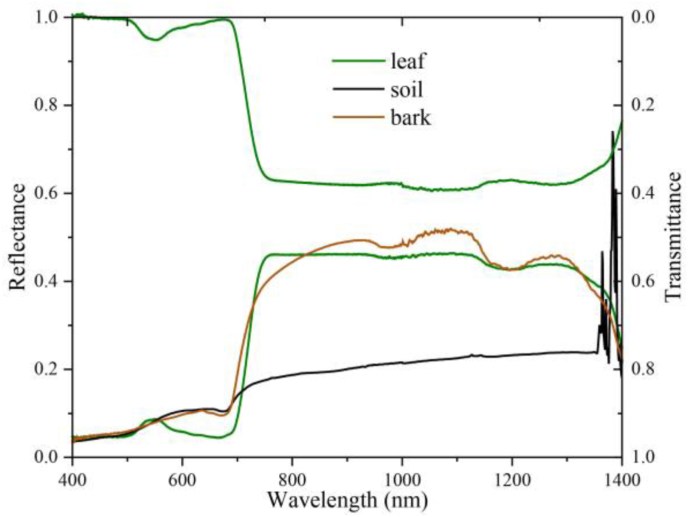
Field measurements of the leaf, soil and bark spectra.

In a controlled dark room setting, an ASD spectrometer (FieldSpec®4 Hi-Res NG) with an illumination collimator and an integrating sphere was used to acquire the leaf reflectance and transmittance. Five apple leaves were measured to determine the leaf reflectance and transmittance. For each leaf, the reflectance and transmittance were measured ten times, and the average of all the leaf measurements was used as the final leaf optical property. Branch reflectance was measured after destructively cutting the bark from the branches and bringing it to the darkroom. The ASD fiber optic probe was fixed to a reflectance probe holder and illuminated with two halogen lamps, and the final reflectance spectra were obtained by averaging 10 measurements of the bark using a white spectral panel (with a reflectance of 98 ​%) to convert the raw brightness to reflectance. The soil spectra were measured at several points in the orchard and finally averaged to obtain the final soil reflectance spectrum. The optical properties of the leaves, bark and ground soil are shown in Fig. 2.

#### 2.1.5 UAV multispectral flight mission

The UAV multispectral imagery was acquired on August 31, 2021, with the DJI Phantom 4 multispectral quadcopter platform. The platform consists of one RGB lenses and five wide-band spectral lenses (Table 1). We carried out the mission at noon when direct solar radiation was dominant. Prior to the mission, the UAV was set to fly at a speed of 2.4 ​m/s and an altitude of 30 ​m, maintaining 80 ​% overlap for both the heading direction and the side direction. Postflight image processing tasks such as photo alignment, image stitching and orthophoto generation were executed using Terra v2.3.3 software (DJI, Shenzhen, China).

**Table 1:** UAV platform parameters.

Parameters	Values
Band 1 (BLUE)	450 ​nm ± 16 ​nm
Band 2 (RED)	560 ​nm ± 16 ​nm
Band 3 (GREEN)	650 ​nm ± 16 ​nm
Band 4 (Red-edge)	730 ​nm ± 16 ​nm
Band 5 (Near-infrared)	840 ​nm ± 16 ​nm
FOV	62.7°
Focal length	5.74 ​mm

### 2.2 Methods

**Fig. 3:**
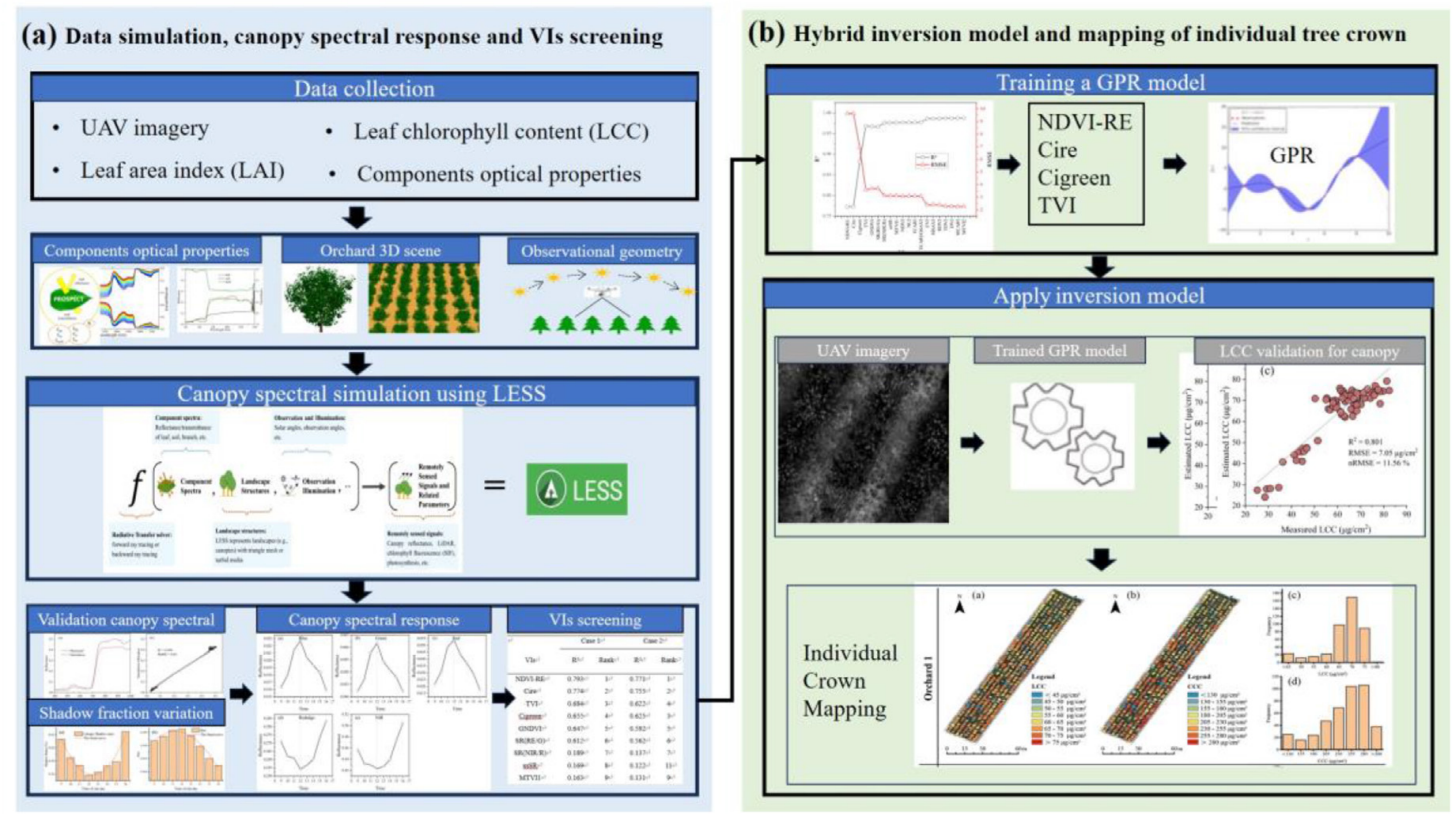
Chlorophyll content retrieval framework. (a) Canopy reflectance simulation using LESS to analyze the canopy spectral response and the resistance of VIs to canopy shadows; (b) construction of the LCC hybrid inversion model and mapping.

Fig. 3 illustrates the retrieval framework for the LCC of individual apple trees. First, we constructed a realistic orchard scene and used the LESS model to simulate canopy spectra at different shadow proportions. Next, we analyzed the canopy spectral response to shadows and evaluated the resistance of various VIs to shadow effects. Subsequently, an iterative strategy was employed to select VIs for LCC retrieval, and a hybrid retrieval model was developed. The real canopy reflectance was subsequently input to the hybrid model to retrieve the LCC of individual tree canopies. Finally, we assessed the accuracy of LCC retrieval and mapped the LCC for individual trees.

#### 2.2.1 3D radiative transfer model and scenario construction

**Fig. 4:**
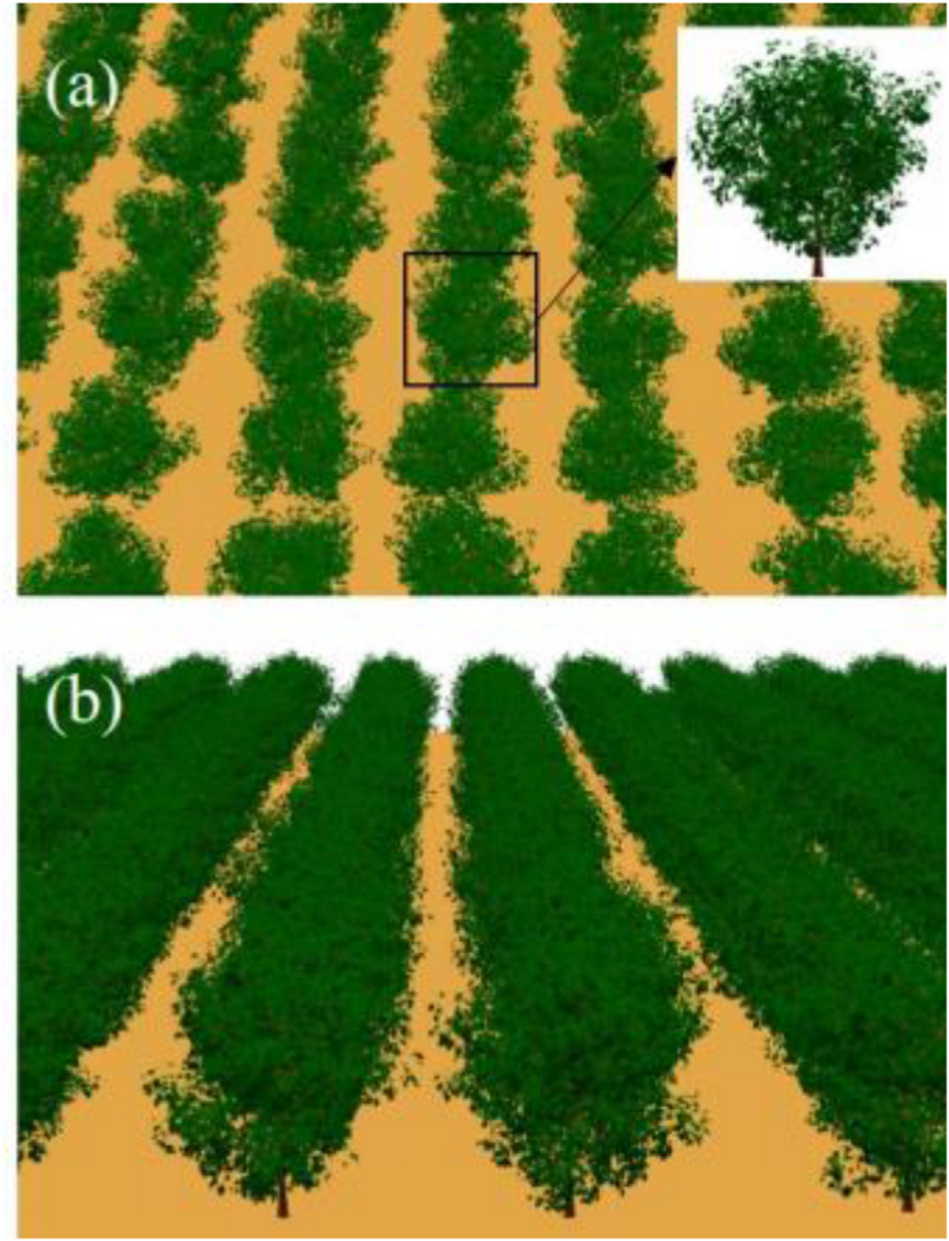
Apple orchard scene constructed by LESS. (a) Top view; (b) front view.

The LESS model is a 3D RTM developed on the basis of ray tracing theory to simulate radiative transfer processes in complex scenes [28]. The user correctly and efficiently simulates the reflectance, spectral images, LiDAR point clouds, etc., by inputting defined 3D representations, solar and observational geometry, and component optical properties. Scenes can range from simple structures to highly detailed 3D objects, and canopy parameter inversion is possible through coupling with the PROSPECT model [43]. Real 3D models of apple trees were created using Onyxtree software (https://www.onyxtree.com/). On the basis of the distribution of apple trees in the orchard, the 3D scene of the orchard was defined as a row structure by means of individual tree replication (Fig. 4).

##### 2.2.1.1 Four-component canopy data and canopy fesc simulation at different times

To quantitatively describe the response of individual tree canopy spectra to intracanopy shadow effects, we need to characterize the variation patterns of canopy shadows. Therefore, we simulated the four-component image data and the fesc of the canopy at the zenith observation angle using LESS. Specifically, we fixed the observation angle of the sensor to (0°, 180°) and calculated the sun position at different moments from 9:00 to 16:00 to characterize the effects of different sun positions on the variation in the canopy shadow fraction. In the simulated four-component image, we assumed that only sunlit and shaded leaves were present in the canopy, with no soil background. Additionally, each pixel was considered to be sunlit if the proportion factor of sunlit leaves was ≥50 ​%. In addition, we simulated the canopy fesc in the near-infrared band at different moments. The shadow portion of the canopy is caused mainly by the scattering of photons, so it follows from the definition of fesc that the larger the proportional portion of the canopy shadow is, the more photons that undergo scattering, which leads to a smaller fesc [36]. That is, by inverse reasoning, the size of the proportion of the shadow can be characterized by the canopy fesc.

##### 2.2.1.2 Canopy reflectance simulation for LCC and CCC inversion

To perform chlorophyll content inversion, we set up a series of reasonable parameters in LESS that can be categorized into three main types of input parameters: leaf structure and chemical parameters, canopy parameters and observation conditions, and optical properties of scene components (shown in Table 2). The leaf structure and chemical parameter inputs include the leaf structure parameter (N), equivalent water thickness (C_w_, μg/cm^2^), brown pigments (C_bp_, μg/cm^2^), carotenoid content (C_xc_, μg/cm^2^), chlorophyll content (C_ab_, μg/cm^2^), and dry matter content (C_m_, μg/cm^2^), which are used to drive the PROSPECT-5 model to yield the leaf directional−hemispherical reflectance and transmittance from 400 ​nm to 2500 ​nm. We set up the leaf inputs according to previous studies [30,44]. We calculated the solar zenith and azimuth angles for different moments of the day at which the data were collected on the basis of the geographic location and the time of day at which the data were collected. The optical properties of the other scene components were set according to field measurements. On the basis of these parameter settings, we generated 8 ​× ​70 ​× ​4 ​× ​9 ​= ​20160 canopy spectra for each moment. For the 8 whole moments from 9:00 a.m. to 16:00 p.m., a total of 8 ​× ​20160 ​= ​161280 data points were generated.

**Table 2:** Input parameters for PROSPECT-5 and LESS.

Type of parameters	Parameters	Unit	Values range
Leaf (PROSPECT-5)	N	–	1∼3
	C_ab_	μg/cm^2^	20∼70
	C_xc_	μg/cm^2^	Depended-Cab
	C_m_	μg/cm^2^	0.01
	C_w_	μg/cm^2^	0.01∼0.03
Canopy	LAI	m^2^/m^2^	0.5∼7
	Time	–	9:00∼16:00
	Solar zenith angle	degrees	Depended-Time
	Solar azimuth angle	degrees	Depended-Time
	View angle	degrees	nadir
Scene optical properties	Background	–	Measurements
	Bark	–	Measurements
	Leaf	–	Depended-PROSPECT-5

#### 2.2.2 Vegetation indices

One of the aims of this work was to check the sensitivity of various UAV broad-band VIs to changes in canopy shadow ratios, so we first sampled the canopy spectra from the LESS simulation in the UAV broadband and calculated the vegetation indices for the field-acquired and simulated reflectances. We identified these commonly used VIs on the basis of previous studies [45,46], and their specific calculation formulas are provided in Table S1.

#### 2.2.3 Analysis of the resistance of VIs to canopy shadows

To identify the VI appropriate for estimating the chlorophyll content from the simulated datasets, an empirical regression model was developed, with the spectral index as the independent variable (x) and the chlorophyll content as the dependent variable (y). By subsequently ranking the correlation coefficients R^2^ of the curve-fit models for each dataset in ascending order, the higher ranked VIs were less sensitive to shadows. Finally, the rankings of the same VI at different canopy shadow fractions were cumulative, and the smaller the result obtained was, the more resistant the VI was to canopy shadow changes.

#### 2.2.4 Construction and validation of a chlorophyll content retrieval model resistant to shadow interference

GPR is a kernel-based probabilistic model for estimating vegetation parameters that has been recognized as more robust than many other machine learning algorithms [18]. GPR is straightforward and easy to understand. It can achieve high accuracy with small sample datasets and can provide a nonparametric relationship between spectral information and the target variable. A comparison between GPR and other methods, such as support vector regression (SVR), kernel ridge regression (KRR), and artificial neural networks (ANNs), demonstrates that GPR not only outperforms other machine learning methods in terms of accuracy but also significantly improves computational efficiency [47,48]. GPR computes posterior distributions on an array of observations and their prior distributions following a Gaussian process.

In this study, we use a three-step approach for LCC and CCC inversion. (1) LCC inversion. Modeling and validation were carried out using LESS simulation data, and the VIs required for inversion were determined by an iterative approach. For each iteration, we divided the dataset into training and validation sets at a 7:3 ratio. Then, on the basis of the determined VIs and corresponding LCC, we trained the GPR-based LCC inversion model using simulation reflectance. The real individual tree canopy reflectance was input into the trained inversion model to retrieve the individual tree LCC, and the accuracy was validated using the measured LCC. Notably, the iterative operations performed here are based on the ranking results of VIs in terms of their resistance to shadow effects. We hypothesize that using VIs with strong resistance to shadow effects as feature inputs for the model can help in constructing a shadow-resistant hybrid inversion model for chlorophyll content. (2) Individual tree LAI estimation. VIs for LAI estimation, namely, NDVI, MTVI2, OSAVI, NDVIre, Cire, and Cigreen, were chosen from the literature and studies([49]; A. [38,50]). The GPR model was calibrated via the measured LAI and calculated VIs with the ultimate goal of predicting the LAI of all the apple trees in the orchard. (3) CCC estimation. The LCC was multiplied by the LAI to yield the CCC.

#### 2.2.5 Evaluation procedures

The accuracy of the inversion results was assessed using various statistics, namely, the coefficient of determination (R^2^), root mean squared error (RMSE), and relative RMSE, which is the ratio of the RMSE to the average of the references, to provide additional evaluation insights.

## 3 Results

### 3.1 Canopy shadow ratio variation and spectral response patterns

#### 3.1.1 Validation of the simulated canopy reflectance

**Fig. 5:**
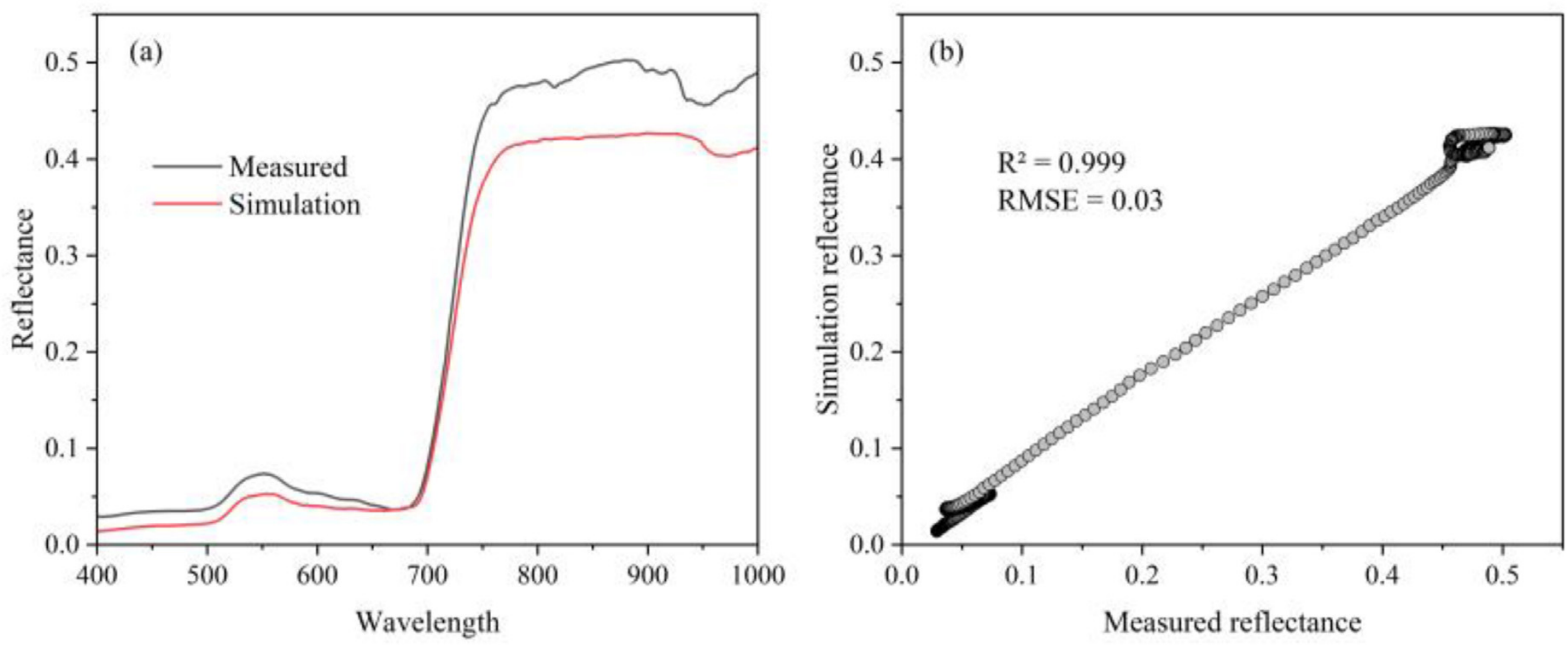
Comparison of simulated and measured individual tree canopy reflectances.

To explain the canopy spectral response to shadows, the reliability of the simulated spectra needs to be verified first. We verified the canopy reflectance obtained from the simulation against the field measurements. The simulated and measured spectral curves at 400–1000 ​nm have similar shapes (Fig. 5 (a)), and they have an R^2^ ​= ​0.999 and an RMSE ​= ​0.03 (Fig. 5 (b)), indicating that the simulated canopy reflectance has high accuracy and can be used for subsequent analysis.

#### 3.1.2 Canopy shadow fraction and canopy fesc at different times in one day

**Fig. 6:**
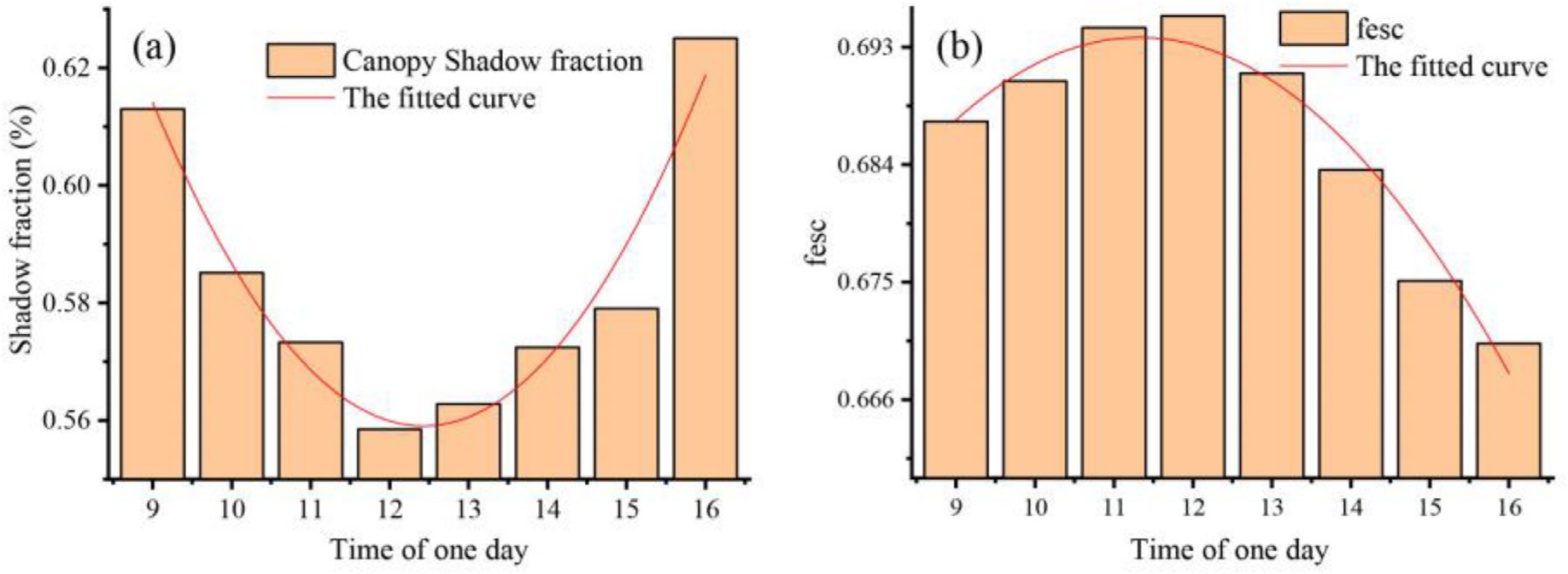
Temporal variation in canopy shadow fraction and fesc.

We calculated the canopy shaded pixel fraction at different moments during the day from the simulated four-component images (Fig. 6(a)), and the canopy shadow proportion demonstrated a parabolic tendency to decrease and then increase with time during the day. At noon, the canopy shadow fraction observed in the nadir direction was the smallest. That is, the closer the time was to noon, the smaller the solar zenith angle when the minimum shadow ratio is obtained from nadir direction observations. Therefore, the best time to use UAVs to acquire data is midday[51] when the proportion of canopy shadow observed is minimized.

Furthermore, the results of the canopy fesc simulation at different moments are shown in Fig. 6(b). With varying time, the canopy fesc tends to increase and then decrease; i.e., the canopy shadow fraction decreases and then increases, and the canopy fesc is greatest and the canopy shadow fraction is smallest at noon.

#### 3.1.3 Analysis of the canopy spectral response caused by variations in the shadow fraction

**Fig. 7:**
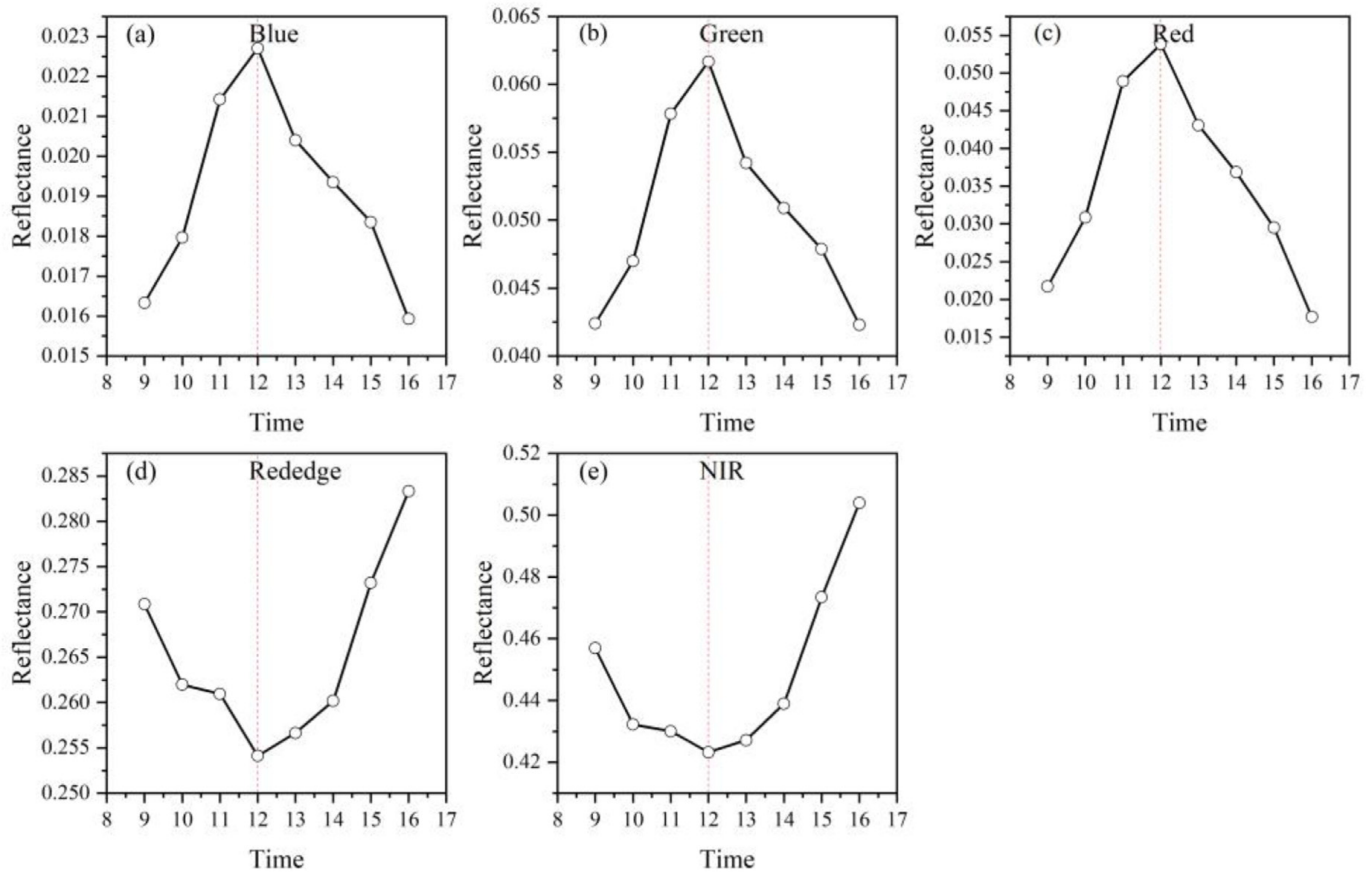
Visible and near-infrared band response to shadows.

We simulated multispectral images of the canopy at different times, as shown in Fig. S2. As shown in the figure, the simulated image shows that the shadow cast by the tree canopy is almost negligible at approximately noon. The position of the sun not only shows a seasonal cycle throughout the year but also varies with time during the day. The solar zenith angle is the smallest at noon and increases with increasing time difference from noon. Consequently, the shadow cast by the canopy varies, as evidenced by the fact that the smaller the solar zenith angle is, the smaller the shadow cast, and the larger the solar zenith angle is, the larger the shadow cast. Furthermore, we extracted the reflectances of individual tree crowns and analyzed the trend of the reflectances in different bands over time during the day (as shown in Fig. 7). In the visible wavelength band, the reflectance first increased but then decreased with time (Fig. 7 (a), (b), (c)). That is, the reflectance reached its maximum value when the canopy shading scale fraction was the smallest. The red edge and near-infrared bands, on the other hand, showed a trend opposite that of the visible band (Fig. 7 (d)(e)).

### 3.2 Analysis of VIs resistance to canopy shadows and VIs determination for hybrid inversion

#### 3.2.1 Analysis of VIs resistance to canopy shadows

Changes in the time of day and solar position cause variations in the crown shadow fraction, which causes variability in the reflectance and VIs. Such variations cause uncertainty in the inversion of canopy parameters. Therefore, we used simulated canopy albedo data to establish empirical relationships between individual VIs and chlorophyll content. We fitted all the simulated data with different canopy shadow fractions and ranked the fitted R^2^; the higher the R^2^ was, the higher the accuracy of the inversion of chlorophyll content for that VI. As shown in Table 3, each case indicates the simulation at different shadow fractions in the canopy. The final rank is obtained by summing the ranks at different shadow fractions, and the smaller the final rank is, the less sensitive the VIs are to the variation in the crown shadow fraction. The top five VIs insensitive to changes in the canopy shadow fraction are NDVI-RE, Cire, Cigreen, TVI, and GNDVI.

**Table 3:** R^2^ values for estimating the chlorophyll content in different shadow scenarios. Cases 1 to 8 represent scenarios with different canopy shadow proportions simulated by LESS, corresponding to the scenarios described in Sections 2.2.1, 3.1.

-	Case 1		Case 2		Case 3		Case 4		Case 5		Case 6		Case 7		Case 8		–
VIs	R^2^	Rank	R^2^	Rank	R^2^	Rank	R^2^	Rank	R^2^	Rank	R^2^	Rank	R^2^	Rank	R^2^	Rank	Summed-rank
NDVI-RE	0.79	1	0.77	1	0.81	1	0.81	1	0.8	1	0.79	1	0.82	1	0.84	1	8
Cire	0.77	2	0.76	2	0.8	2	0.8	2	0.79	2	0.77	2	0.8	2	0.83	2	16
TVI	0.68	3	0.62	4	0.62	5	0.6	6	0.61	6	0.65	4	0.66	4	0.75	3	35
Cigreen	0.66	4	0.63	3	0.7	3	0.69	3	0.68	3	0.68	3	0.69	3	0.72	4	26
GNDVI	0.65	5	0.59	5	0.63	4	0.64	4	0.64	4	0.65	5	0.65	5	0.72	5	37
SR(RE/G)	0.61	6	0.56	6	0.62	6	0.62	6	0.62	6	0.62	6	0.63	6	0.68	6	46
SR(NIR/R)	0.19	7	0.14	7	0.15	10	0.15	10	0.16	9	0.17	9	0.2	7	0.3	7	66
mSR	0.17	8	0.12	11	0.14	12	0.14	12	0.15	11	0.15	10	0.18	8	0.28	8	70
MTVI1	0.16	9	0.13	9	0.15	10	0.14	11	0.15	10	0.14	11	0.14	9	0.19	10	79
NDVI	0.12	10	0.09	12	0.12	13	0.12	13	0.12	13	0.12	12	0.13	11	0.2	9	83
NLI	0.12	11	0.09	13	0.11	15	0.1	15	0.11	14	0.11	13	0.11	13	0.17	11	105
TCARI	0.11	12	0.13	9	0.42	7	0.35	7	0.28	7	0.2	7	0.13	10	0.14	13	72
TCARI/OSAVI	0.11	13	0.12	10	0.35	8	0.25	8	0.25	8	0.19	8	0.13	12	0.13	16	83
OSAVI	0.11	14	0.08	15	0.11	14	0.1	14	0.11	15	0.11	15	0.11	14	0.15	12	113
EVI	0.1	15	0.08	14	0.1	16	0.1	15	0.1	16	0.1	16	0.1	15	0.13	15	122
MSAVI	0.1	16	0.08	16	0.09	17	0.09	17	0.09	17	0.09	17	0.09	16	0.13	14	130
RDVI	0.09	17	0.07	17	0.09	18	0.09	18	0.09	18	0.09	18	0.08	17	0.12	17	138
TDVI	0.07	18	0.06	18	0.08	19	0.07	19	0.08	19	0.07	19	0.06	19	0.08	18	149
DVI	0.05	19	0.05	20	0.06	20	0.06	20	0.06	20	0.05	20	0.04	20	0.045	20	158
MCARI	0.03	20	0.05	19	0.2	9	0.15	9	0.12	12	0.11	14	0.06	18	0.01	21	122
MTVI2	0.01	21	0.02	21	0.05	21	0.04	21	0.04	21	0.03	21	0.02	21	0.01	20	167

#### 3.2.2 VIs for hybrid inversion

**Fig. 8:**
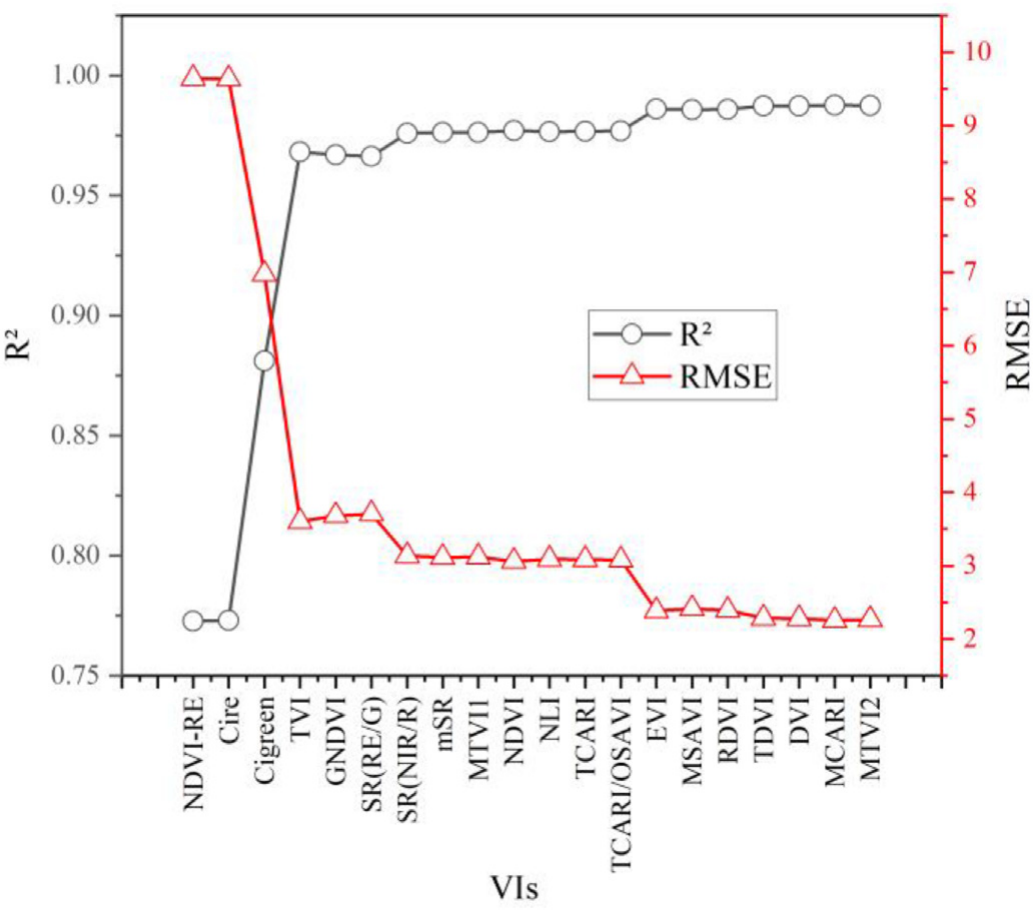
VI determination for chlorophyll content inversion.

In the previous section, we ranked the VIs that are insensitive to changes in the canopy shadow fraction, and further, when we utilize these VIs for the inversion of canopy LCC, we must determine how many VIs to use, which results in the highest inversion accuracy. Therefore, we performed an iterative operation on these VIs, which was implemented in the following steps: first, the first-ranked VI was used as the base VI for modeling and validation, and the next-ranked VIs was added sequentially. For each VI added, modeling and validation were performed until the validation accuracy was minimized, at which point the iteration was stopped. The four vegetation indices, i.e., the NDVI-RE, Cire, Cigreen, and TVI indices, are almost enough to achieve the best inversion accuracy (Fig. 8).

#### 3.2.3 Comparison of simulated and measured VIs

**Fig. 9:**
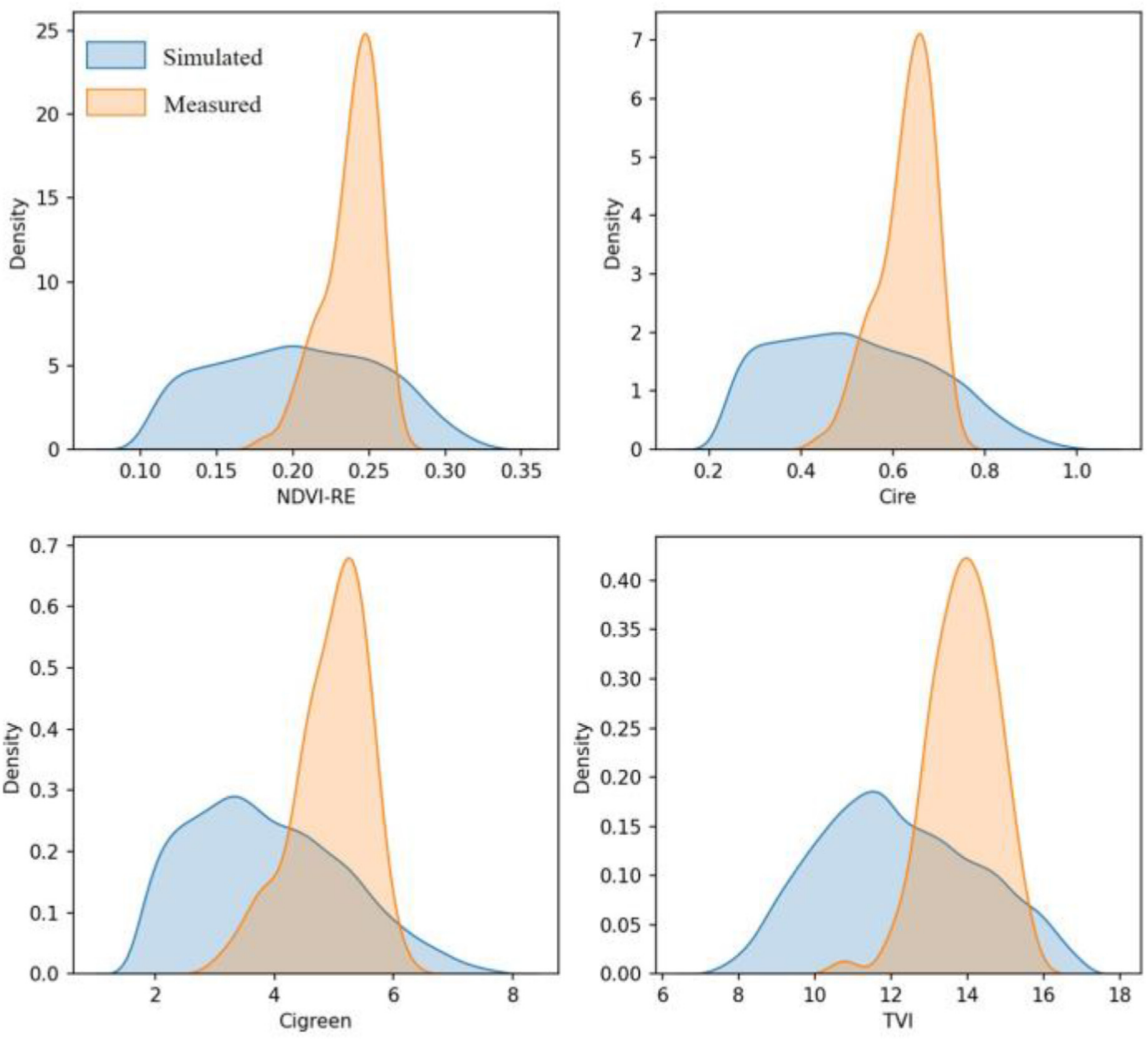
Distribution of the simulated and measured VIs.

We compared four VIs calculated and screened for modeling from the simulated canopy reflectance and UAV-measured reflectance. The simulated and measured data had similar distributions, and the simulated data had a wider distribution range (Fig. 9). Therefore, the simulated dataset can provide considerable training data for modeling and can enable LCC retrieval for individual trees.

### 3.3 Performance of the shadow-resistant chlorophyll content inversion method for individual trees

**Fig. 10:**
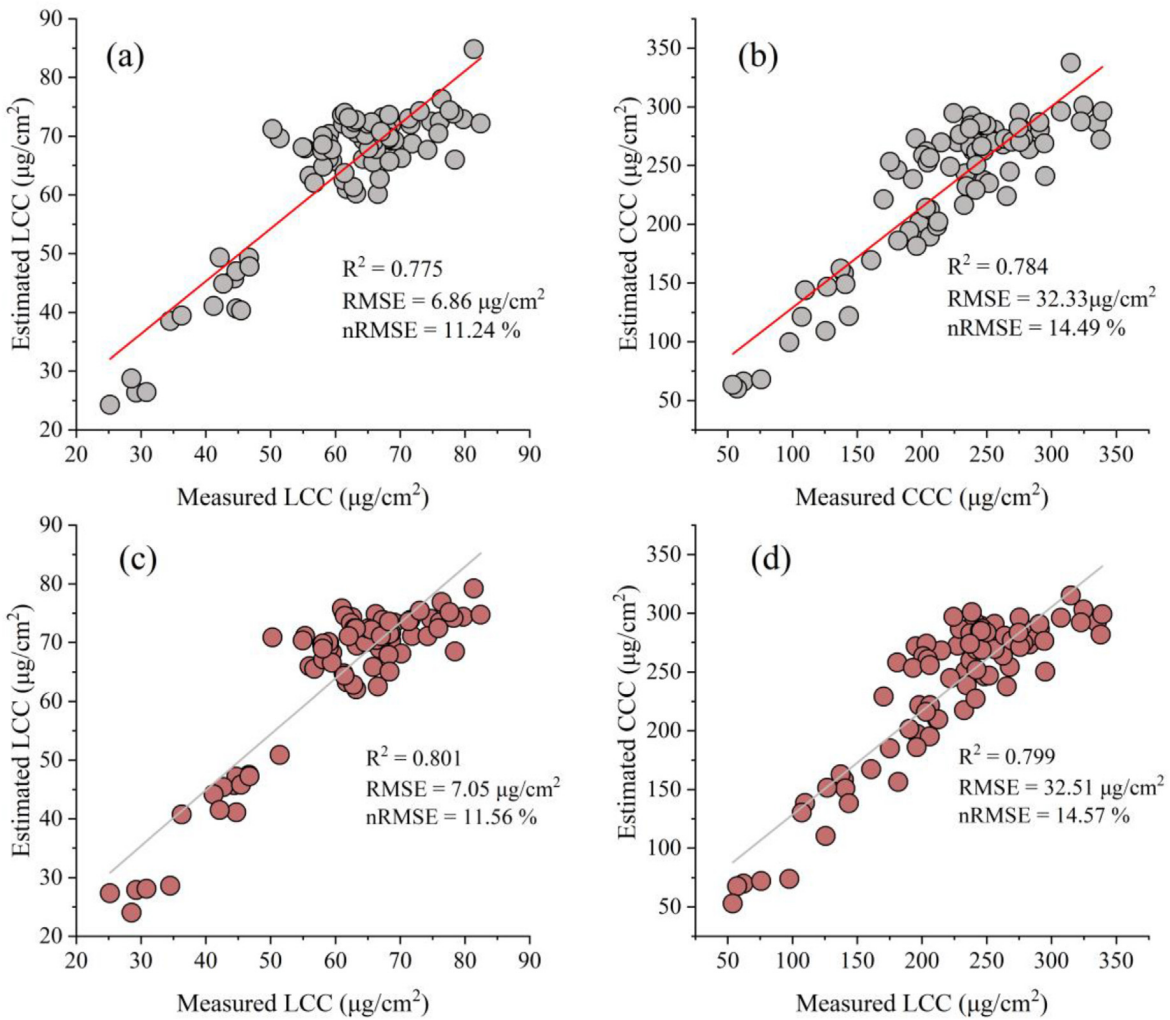
Comparison of the estimated LCC and CCC results with the measured values. (a) and (b) Results of the inversion strategy developed in this study; (c) and (d) inversion results after the removal of canopy shadow pixels.

We developed a hybrid LCC inversion model for GPR and VIs. Compared with the measurements, the inverted LCC yielded an R^2^ ​= ​0.78, an RMSE ​= ​6.86 ​μg/cm^2^, and an nRMSE ​= ​12.24 ​% (e.g., Fig. 10 (a)). However, poor estimation accuracy is encountered at higher values of LCC (LCC >60 ​μg/cm^2^) due to spectral saturation. Furthermore, we estimated the CCC by multiplying the LCC obtained from the inversion with the LAI obtained by calibrating the GPR model. Compared with the measurements, the estimated CCC showed an R^2^ ​= ​0.78, an RMSE ​= ​32.33 ​μg/cm^2^, and an nRMSE ​= ​14.49 ​% (Fig. 10 (b)). Although the correlation coefficient of the CCC was comparable to that of the LCC, the nRMSE was lower, and the error transfer due to multiplication may be the main reason for the decrease.

To further illustrate the feasibility of our method, we applied a threshold method to remove canopy shadow pixels on the basis of our previous research [32], using only sunlit pixels for LCC and CCC inversion (Fig. 10 c, d). Notably, apart from masking shadow pixels in the original data, the remaining inversion steps were consistent with the method proposed in this study. The results revealed that, after shadow pixels were removed, the coefficient of determination (R^2^) was slightly greater than that of the method in this study (LCC: 0.80 vs. 0.78; CCC: 0.80 vs. 0.78), whereas the validation accuracy was slightly lower than that of the method developed in this study (LCC: 7.05 ​μg/cm^2^ vs. 6.86 ​μg/cm^2^ (RMSE), 11.56 ​% vs. 11.24 ​% (nRMSE); CCC: 32.51 ​μg/cm^2^ vs. 32.33 ​μg/cm^2^ (RMSE), and 14.57 ​% vs. 14.49 ​% (nRMSE)). This further demonstrates the effectiveness of our shadow-resistant chlorophyll inversion method for individual apple trees in a canopy.

### 3.4 Spatial distribution mapping of LCC and CCC

**Fig. 11:**
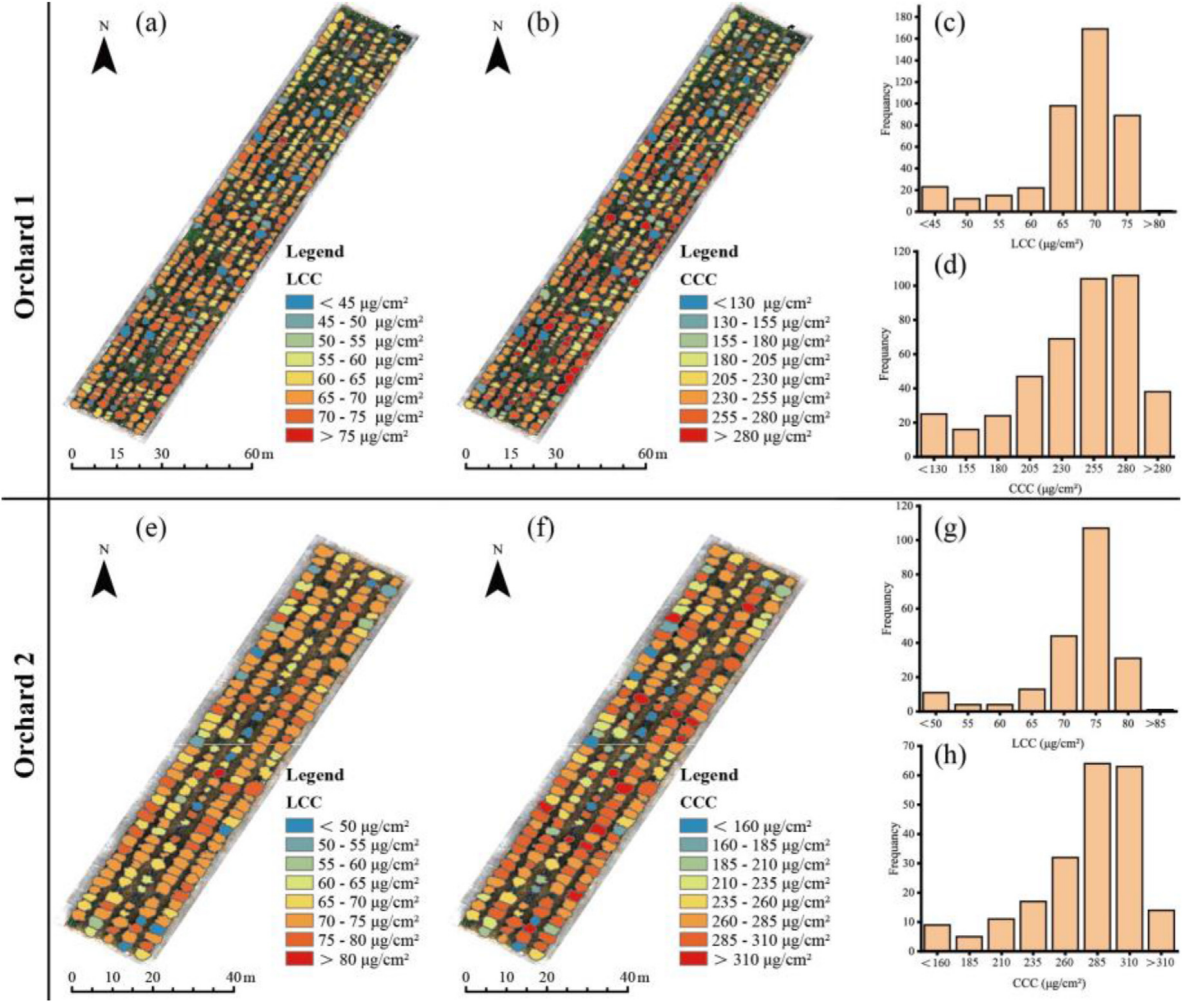
Spatial mapping and statistics for the LCC and CCC in two orchards.

The LCC and CCC were retrieved for all single trees from two apple orchards via the developed model (orchard 1: 429 trees; orchard 2: 215 trees) and were spatially mapped (Fig. 11). The diversity and spatial heterogeneity of the LCC and CCC among single trees in the two orchard scenarios demonstrated the ability of high-resolution UAV multispectral imagery to characterize the spatial variability of individual tree canopy parameters. Furthermore, we counted the LCC and CCC of individual trees in two orchards, and their values showed a normal distribution, which may support a priori knowledge for more accurate inversion. For orchard 1, the LCC was distributed mainly between 45 and 80 ​μg/cm^2^ (Fig. 11 (d)), and the CCC was distributed mainly between 130 and 280 ​μg/cm^2^ (Fig. 11 (c)). For orchard 2, the LCC was distributed mainly between 50 and 80 ​μg/cm^2^ (Fig. 11 (g)), and the CCC was distributed mainly between 160 and 310 ​μg/cm^2^ (Fig. 11 (h)). The estimates for Orchard 2 were all slightly higher than those for Orchard 1.

## 4 Discussion

### 4.1 Response of individual tree canopy spectra and VIs to intercanopy shadow effects

This study quantitatively described the variation in internal shadows within individual tree canopies by simulating centimeter-scale images of the canopy at different times. Furthermore, the response of canopy spectra to shadow variations was analyzed. The results indicate that when ultrahigh-resolution spectral images are used for inversion, the internal shadow effects of the canopy cannot be ignored. Most notably, the proportion of canopy shadows is closely related to the shape and structure of the canopy in addition to the solar and observational geometry. Although this study conducted simulation experiments on canopies with the same shape and structure, overall, the variation in canopy shadow effects observed corresponds to the findings of [36]. Therefore, our series of simulations and hypotheses are considered reasonable and can be used to explain the variations in shadow effects and spectral responses in individual trees with different structures and canopy shapes.

**Fig. 12:**
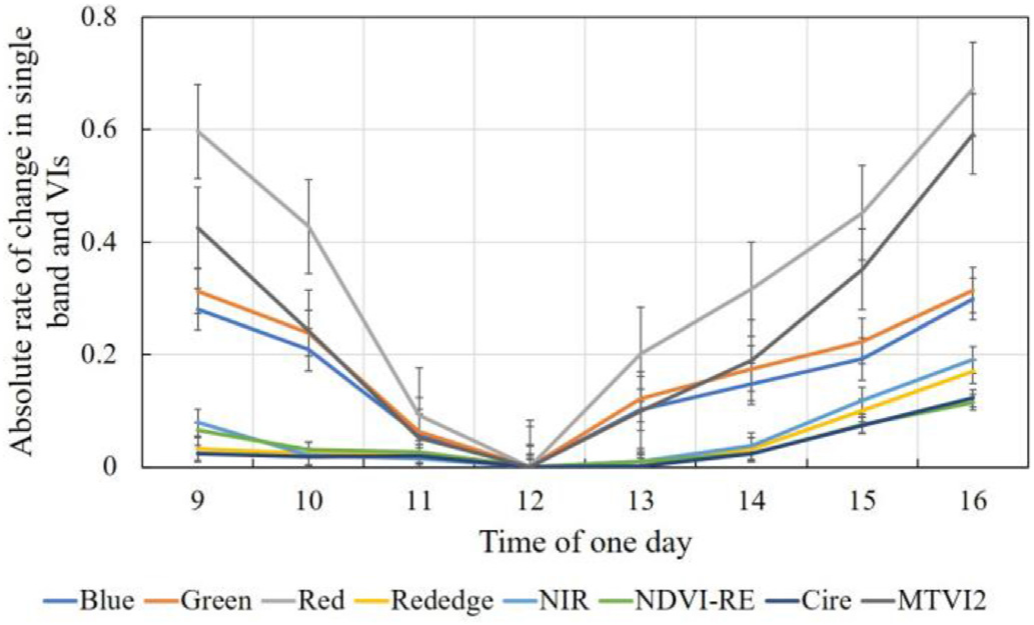
Absolute rates of change in single-band reflectance and vegetation indices at different canopy shadow fractions. The calculations are based on the time with the smallest canopy shadow fraction (12:00) as the reference.

Using the minimum canopy shadow proportion as a baseline, we calculated the rate of change in canopy spectra at different shadow proportions and various times of the day (Fig. 12). Compared with changes in the visible light spectrum, the changes in the near-infrared bands are negligible, especially near the baseline point (e.g., 10:00–14:00). In high-resolution remote sensing images, shadow pixels are often artifacts caused by scattering from neighboring pixels. Therefore, as the canopy shadow proportion increases, more scattered light is captured by the sensor, leading to greater variability in canopy reflectance, especially in the visible spectrum. The difference between the reflectances of shadow pixels and their true reflectances can obscure important spectral features, leading to erroneous estimations of the target parameters. Specifically, the estimation of LCC relies on the reflectance in the visible light spectrum, and it can be inferred that a larger canopy shadow proportion will result in greater precision loss in LCC estimation.

VIs, as mathematical operations on single bands, can be used as substitutes for the single-band reflectance in LCC inversion. While VIs can mitigate the influence of interfering factors to some extent, their resistance to canopy shadows, a factor that has not been addressed in previous studies, must be evaluated. By simulating different canopy shadow patterns using a 3D RTM, we achieve this objective. In the inversion of the canopy LCC using high-resolution UAV spectra, shadow pixels are generally removed through masking algorithms, and only sunlit pixels are used for inversion. This method relies on the robustness of the masking algorithm. Therefore, we aimed to develop an LCC inversion framework that does not depend on masking algorithms to resist shadow interference. This framework first requires the analysis of spectral responses to canopy shadows, as well as the resistance of the VIs used for modeling to shadows. Similarly, we calculated the absolute rates of change for several VIs at different canopy shadow fractions (Fig. 12). The absolute rates of change for the NDVI-RE and Cire data were relatively lower than those for the single-band reflectance data, whereas the MTVI2 data exhibited greater variations. Thus, our approach for selecting VIs is reasonable. Through iterative screening, we identified VIs that are insensitive to canopy shadow changes as feature inputs for LCC retrieval, effectively reducing the accuracy loss caused by shadows. Our results show that the proposed method is effective, with an accuracy comparable to or even better than that achieved by inverting using only sunlit pixels (Fig. 9). To some extent, we simplified the traditional LCC inversion process for individual trees in a canopy by eliminating the need to mask shadow pixels.

### 4.2 Uncertainty, potential sources of error, and improvement strategies

**Fig. 13:**
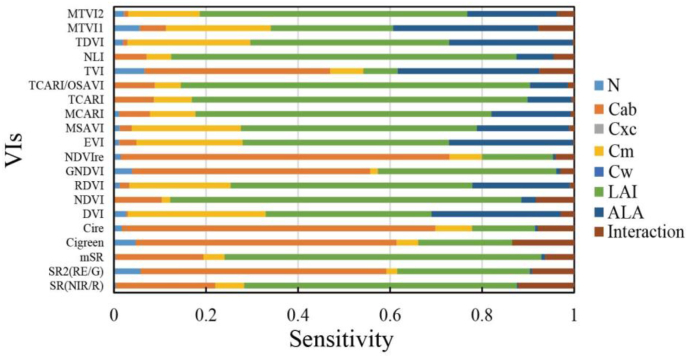
Analysis of VI sensitivity to disturbance factors.

We further discuss the uncertainties and influencing factors of the inversion method we developed. For the LCC, the uncertainties stem primarily from the selected VIs. Theoretically, indices chosen for estimating canopy parameters should be highly sensitive to target covariates while remaining unaffected by disturbance factors. That is, these VIs should be insensitive to canopy shadow effects and other variables in addition to being sensitive to the LCC. Notably, in the previous section, we demonstrated the resistance of the screened VIs to shadow effects, and further information on their sensitivity to other confounders is needed. With this foundation, we simulated UAV broadband reflectance by considering various leaf physiological and biochemical traits, geometric attributes, canopy structural parameters, and background factors. We then calculated the selected VIs individually, examined their variation patterns, and assessed their effectiveness in estimating canopy parameters. In this study, 20 commonly used broad-band VIs were analyzed for their resistance to canopy shadows and screened by iterative optimization of the VIs used for modeling and validation. However, these VIs, while being sensitive to canopy chlorophyll content and resistant to canopy shadows, should also be insensitive to disturbance factors. We further analyzed the sensitivity of these VIs to disturbance factors other than LCC, as shown in Fig. 13. Among all disturbance factors, the VIs with the greatest sensitivity to chlorophyll content were NDVI-RE, Cire, Cigreen, TVI, GNDVI, and SR2 (RE/G). These VIs correspond to the top six VIs in Section 4.2.1. Although the GNDVI and SR2 (RE/G) are more sensitive to the chlorophyll content than the TVI is, they are less resistant to the LAI and canopy shadows than the TVI is. Therefore, VIs that are sensitive to chlorophyll content but not to the LAI or shadows are better selected via our VI screening. However, in the inversion of canopy parameters, the mutual influence between the LCC and LAI is inevitable, leading to significant uncertainties in LCC estimation results. Consequently, many studies have attempted to disentangle the LCC and LAI or to construct LCC VIs that are insensitive to the LAI by utilizing additional spectral bands [52]. However, decoupling them is very difficult. Instead, we determined the VIs to be used for chlorophyll inversion by the hybrid method by iterating over the sorted VIs. This hybrid method using VIs as input features can somewhat improve the accuracy of chlorophyll content estimation when raw band information is used as input. This is because the VI is constructed by mathematically combining single bands, which itself is, to some extent, resistant to disturbing factors such as the environment. Our method can be used as an alternative to decoupled LCC and LAI methods to some extent.

The CCC is the product of the LCC and LAI. Therefore, the uncertainty of the CCC is related to two terms. Sources of LCC uncertainty are discussed in the previous paragraph, and the uncertainty caused by LAI estimation is discussed here. As shown in Fig. S3, the estimated LAI is compared with the real measurements, and the calibrated LAI estimation model has high accuracy, with an R^2^ ​= ​0.68, an RMSE ​= ​0.30 ​m^2^/m^2^, and an nRMSE ​= ​8.48 ​%. Therefore, although the correlation coefficient of the CCC is comparable to that of the LCC, the lower precision is also explained. The error transfer due to the multiplication of the LCC and LAI may be the main reason for the reduced accuracy of the CCC. In future work, constructing an inversion model with dual outputs, namely, LCC and LAI, using a 3D RTM may improve the accuracy of CCC inversion. This is because this approach can guarantee the estimation accuracy of the LCC and LAI at the same time and reduce the error transfer.

Canopy shadows result from a combination of multiple factors, primarily canopy structural effects and observation geometry. Therefore, the hybrid LCC inversion framework we propose for individual tree crowns is also influenced by these two factors. Although we developed a shadow-resistant hybrid LCC inversion method based on a 3D RTM, it is applicable mainly to open-canopy orchards, where the complexity of the tree crowns is relatively low. When canopy complexity is high, such as in forested scenes, this method may require further testing. Research by Ref. [36] shows that as canopy complexity increases, the shadows cast become larger, with VIs being least affected by shadows in the hotspot direction. Therefore, for highly heterogeneous scenes, our hybrid inversion method can be improved by optimizing the observation direction. Recently, the canopy scattering coefficient (CSC) has been regarded an interesting parameter for characterizing canopy structural effects [53,54]. suggested that by combining the CSC with VIs, the impact of canopy structural effects can be effectively reduced, thereby improving the retrieval success rates for the LCC and LAI. Thus, incorporating the CSC into our hybrid inversion framework can further enhance model performance. Additionally, the fusion of multimodal data, which allows 3D RTMs to simulate more realistic canopy spectra, can help train more robust LCC inversion models. For example, the use of LiDAR point clouds to provide structural constraints for canopy spectrum simulations combined with synchronously collected canopy spectra for LCC prediction could further improve the inversion accuracy [55].

### 4.3 Future research directions and application potential

Currently, for most satellite images, which have spatial resolutions ranging from the submeter scale to the kilometer scale, sunlit and shaded vegetation are integral parts of the remote sensing image pixels acquired from Earth observations. Shadows can lead to a reduction in or loss of information in the image, resulting in inaccurate calculated VIs, which in turn leads to more downstream errors from satellite pixel-level biophysical parameter estimation. In this study, on the basis of high-spatial resolution multispectral UAV imagery and a 3D RTM, we assessed the resistance of several VIs to canopy shadows and designed LCC and CCC inversion strategies on the basis of interference-resistant VIs. This provides an idea for minimizing the effect of shadows on canopy parameter inversion at the satellite scale, and the validation of our shadow-resistant hybrid method at the satellite scale is also one of our future studies. Of course, for ultrahigh-resolution spectral images (such as those with centimeter-level resolution), the use of the popular P-theory in recent years to correct the reflectivity of shadow pixels is also an effective way to suppress the shadow effect in canopies [33].

## 5 Conclusion

In this study, we developed a hybrid inversion strategy for LCC estimation in individual apple tree crowns on the basis of a 3D RTM and GPR. This method does not rely on canopy shadow pixel masking algorithms, enabling it to effectively mitigate the impact of shadows. First, we used a 3D RTM to analyze the pattern of the canopy shadow fraction over time in individual apple trees and analyzed the response law of canopy spectra to this pattern. We then evaluated the resistance of the VIs to canopy shadows, screening four VIs used for model construction. Finally, we developed a hybrid inversion model and performed an accuracy evaluation and spatial mapping. The results revealed that the response of individual apple tree crown spectra to shadows exhibited opposing changes in the visible and near-infrared bands. That is, the spectral reflectance increased and then decreased in the visible band, whereas the opposite occurred in the near-infrared band. Moreover, different VIs presented different resistances to shadows, and the screened VIs used for modeling were NDVI-RE, Cire, Cigreen, and TVI. Finally, accuracy validation was performed for individual apple tree crowns (LCC: R^2^ ​= ​0.775, RMSE ​= ​6.86 ​μg/cm^2^, nRMSE ​= ​12.24 ​%; CCC: R^2^ ​= ​0.784, RMSE ​= ​32.33 ​μg/cm^2^, and nRMSE ​= ​14.49 ​%). Finally, the accuracy of the method was validated on individual apple tree canopies. Compared with the results obtained using only illuminated pixels, the results of the proposed approach demonstrated comparable or superior accuracy. We propose a shadow-resistant framework for LCC retrieval, providing insights for more precise retrieval of detailed canopy-level LCC data. Furthermore, we mapped the spatial distributions of the LCC and CCC, which revealed high interspecific heterogeneity between trees. Considering the advantages of the 3D RTM, the responses of the canopy spectra to more canopy structures or observation geometries will be subsequently evaluated, and their impact on the inversion results will be quantitatively characterized.

## Data availability

The authors do not have permission to share data.

## Declaration of competing interest

The authors declare that they have no known competing financial interests or personal relationships that could have appeared to influence the work reported in this paper.
