# Supplementary material for: Retrieving the chlorophyll content of individual apple trees by reducing canopy shadow impact via a 3D radiative transfer model and UAV multispectral imagery
Source: Plant Phenomics. 2025 Mar 6;7(1):100015. doi: 10.1016/j.plaphe.2025.100015 (PMC12710017; doi:10.1016/j.plaphe.2025.100015)
Supplement: Multimedia component 1 [file mmc1.docx]

# Supplementary Material

**Retrieving chlorophyll content of individual apple tree by reducing canopy shadow impact using 3D radiative transfer model and UAV multispectral imagery**

Chengjian Zhang ^a, b^, Zhibo Chen ^a, *^, Riqiang Chen ^a, b^, Wenjie Zhang ^a, b^, Dan Zhao ^b, c^,Guijun Yang ^b,^ Bo Xu ^b^, Haikuan Feng ^b^, Hao Yang ^b, *^

*^1^ School of Information Science and Technology, Beijing Forestry University, Beijing 100083, China.*

*^2^ Key Laboratory of Quantitative Remote Sensing in Agriculture of Ministry of Agriculture and Rural Affairs, Information Technology Research Center, Beijing Academy of Agriculture and Forestry Sciences, Beijing 100097, China.*

* Correspondence: [zhibo@bjfu.edu.cn](mailto:zhibo@bjfu.edu.cn); [yangh@nercita.org.cn](mailto:yangh@nercita.org.cn)


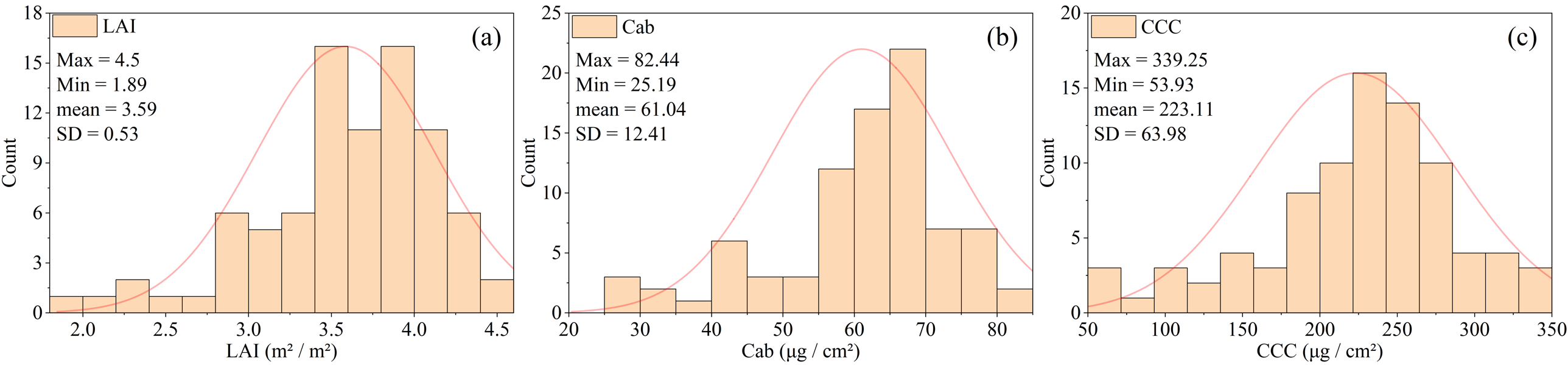


**Fig. S1**. Distribution of measured data. (a) LAI; (b)Cab; (c) CCC.


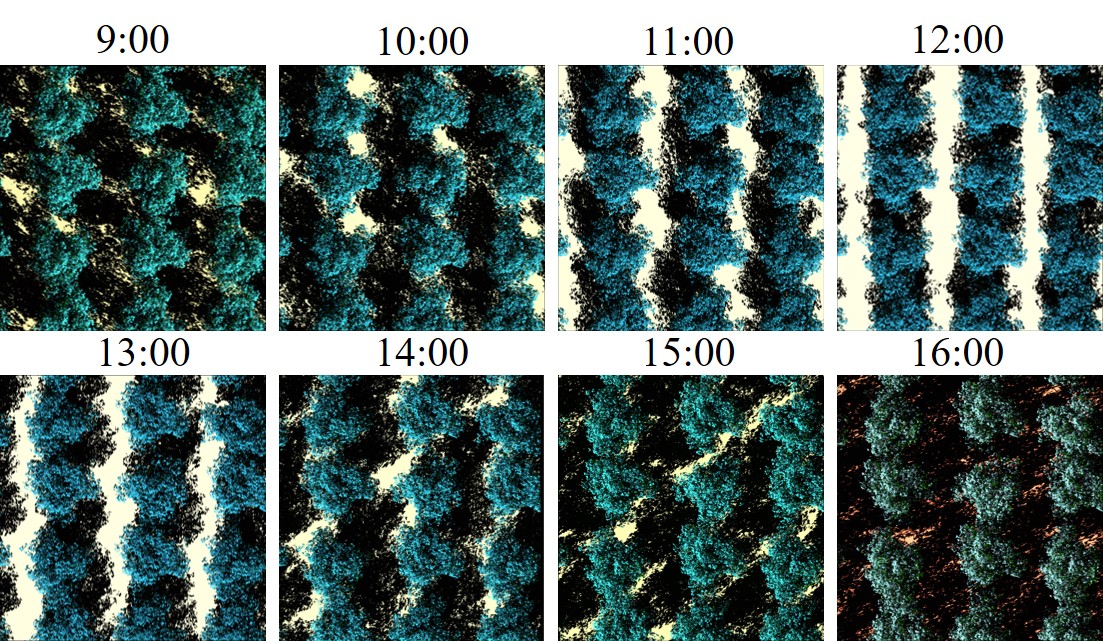


**Fig. S2**. Simulated time-series multispectral images.


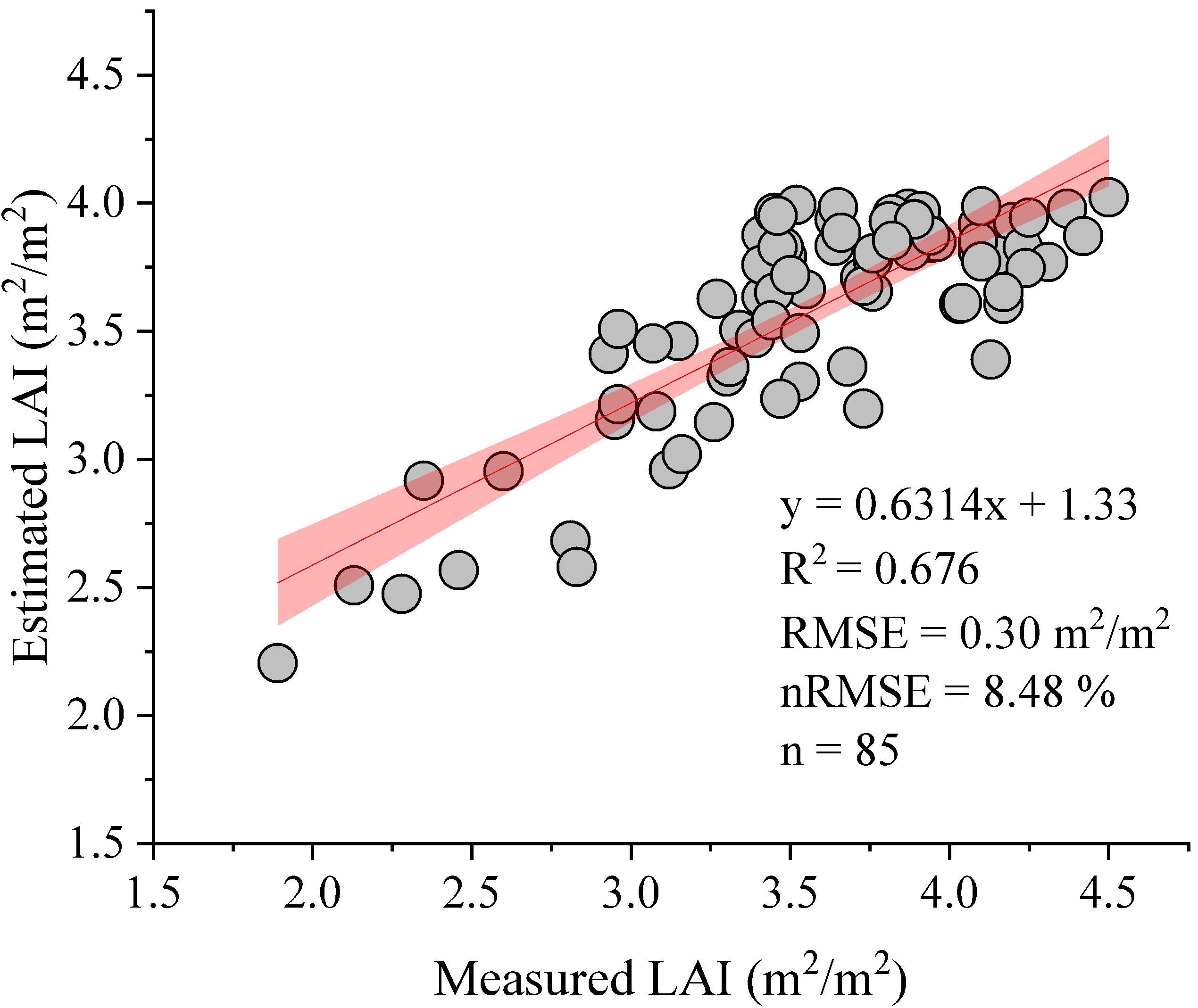


**Fig. S3**. Results of LAI cross-validation.

**Table S1**. VIs for this work.

| VIs | Description | Formula | Reference |
| --- | --- | --- | --- |
| SR 1 | Simple Ratio 1 | $\frac{\rho_{NIR}}{\rho_{red}}$ | (Sims and Gamon, 2002) |
| SR 2 | Simple Ratio 2 | $\frac{\rho_{re}}{\rho_{green}}$ | (CARTER, 1994) |
| mSR | Modified Simple Ratio | $\frac{\frac{\rho_{NIR}}{\rho_{red}}-1}{\sqrt{\frac{\rho_{NIR}}{\rho_{red}}+1}}$ | (Chen, 1996) |
| Cigreen | Green Chlorophyll Index | $\frac{\rho_{NIR}}{\rho_{green}}-1$ | (Gitelson et al., 2003) |
| Cire | Red edge Chlorophyll Index | $\frac{\rho_{NIR}}{\rho_{re}}-1$ | (Gitelson et al., 2003) |
| DVI | Difference Vegetation Index | $\rho_{NIR}-\rho_{red}$ | (Roujean and Breon, 1995) |
| NDVI | Normalized Difference Vegetation Index | $\frac{\rho_{NIR}-\rho_{red}}{\rho_{NIR}+\rho_{red}}$ | (Rouse et al., 1974) |
| RDVI | Renormalized Difference Vegetation Index | $\frac{\rho_{NIR}-\rho_{red}}{\sqrt{\rho_{NIR}+\rho_{red}}}$ | (Roujean and Breon, 1995) |
| GNDVI | Green Normalized Difference  Vegetation Index | $\frac{\rho_{NIR}-\rho_{green}}{\rho_{NIR}+\rho_{green}}$ | (Gitelson et al., 1996) |
| NDVI-RE | Normalized Difference Red-edge Vegetation Index | $\frac{\rho_{NIR}-\rho_{re}}{\rho_{NIR}+\rho_{re}}$ | (Fitzgerald et al., 2006) |
| EVI | Enhanced Vegetation Index | $2.5\frac{\rho_{NIR}-\rho_{red}}{\rho_{NIR}+6\rho_{red}-7.5\rho_{blue}+1}$ | (Huete et al., 1994) |
| MSAVI | Modified Soil-Adjusted Vegetation Index | $\frac{{2\rho}_{NIR}+1-\sqrt{{{(2\rho}_{NIR}+1)}^{2}-8(\rho_{NIR}-\rho_{red})}}{2}$ | (Qi et al., 1994) |
| MCARI | Modified Chlorophyll Absorption Ratio Index | $[\rho_{re}-\rho_{red}-0.2(\rho_{re}-\rho_{green})]\frac{\rho_{re}}{\rho_{red}}$ | (Daughtry et al., 2000) |
| TCARI | Transformed Chlorophyll Absorption in Reflectance Index | $3\left[ \rho_{re}-\rho_{red}-0.2\frac{{(\rho_{re}-\rho_{green})\rho}_{re}}{\rho_{red}} \right]$ | (Haboudane et al., 2002) |
| TCARI/OSAVI | Integrated narrow-band vegetation indices | $\frac{TCARI}{\frac{\rho_{NIR}-\rho_{red}}{\rho_{NIR}+\rho_{red}+0.16}}$ | (Haboudane et al., 2002) |
| TVI | Triangular Vegetation Index | $0.5\left[ {120(\rho}_{NIR}-\rho_{green})-200(\rho_{red}-\rho_{green}) \right]$ | (Rouse et al., 1974) |
| NLI | Non-Linear Vegetation Index | $\frac{{\rho_{NIR}}^{2}-\rho_{red}}{{\rho_{NIR}}^{2}+\rho_{red}}$ | (Goel and Qin, 1994) |
| TDVI | Transformed Difference Vegetation Index | $1.5\frac{\rho_{NIR}-\rho_{red}}{\sqrt{{\rho_{NIR}}^{2}+\rho_{red}+0.5}}$ | (Bannari et al., 2002) |
| MTVI1 | Modified Triangular Vegetation Index 1 | $1.2\left[ {1.2(\rho}_{NIR}-\rho_{green})-2.5(\rho_{red}-\rho_{green}) \right]$ | (Haboudane et al., 2004) |
| MTVI2 | Modified Triangular Vegetation Index 2 | $\frac{1.5\left[ {1.2(\rho}_{NIR}-\rho_{green})-2.5(\rho_{red}-\rho_{green}) \right]}{\sqrt{{{(2\rho}_{NIR}+1)}^{2}-6\rho_{NIR}-5\sqrt{\rho_{red}}-0.5}}$ | (Haboudane et al., 2004) |

# Reference

Bannari, A., Asalhi, H., Teillet, P.M., 2002. Transformed difference vegetation index (TDVI) for vegetation cover mapping, in: IEEE International Geoscience and Remote Sensing Symposium. pp. 3053–3055 vol.5. https://doi.org/10.1109/IGARSS.2002.1026867

CARTER, G.A., 1994. Ratios of leaf reflectances in narrow wavebands as indicators of plant stress. International Journal of Remote Sensing 15, 697–703. https://doi.org/10.1080/01431169408954109

Chen, J.M., 1996. Evaluation of Vegetation Indices and a Modified Simple Ratio for Boreal Applications. Canadian Journal of Remote Sensing 22, 229–242. https://doi.org/10.1080/07038992.1996.10855178

Daughtry, C.S.T., Walthall, C.L., Kim, M.S., de Colstoun, E.B., McMurtrey, J.E., 2000. Estimating Corn Leaf Chlorophyll Concentration from Leaf and Canopy Reflectance. Remote Sensing of Environment 74, 229–239. https://doi.org/10.1016/S0034-4257(00)00113-9

Fitzgerald, G.J., Rodriguez, D., Christensen, L.K., Belford, R., Sadras, V.O., Clarke, T.R., 2006. Spectral and thermal sensing for nitrogen and water status in rainfed and irrigated wheat environments. Precision Agric 7, 233–248. https://doi.org/10.1007/s11119-006-9011-z

Gitelson, A.A., Gritz †, Y., Merzlyak, M.N., 2003. Relationships between leaf chlorophyll content and spectral reflectance and algorithms for non-destructive chlorophyll assessment in higher plant leaves. Journal of Plant Physiology 160, 271–282. https://doi.org/10.1078/0176-1617-00887

Gitelson, A.A., Kaufman, Y.J., Merzlyak, M.N., 1996. Use of a green channel in remote sensing of global vegetation from EOS-MODIS. Remote Sensing of Environment 58, 289–298. https://doi.org/10.1016/S0034-4257(96)00072-7

Goel, N.S., Qin, W., 1994. Influences of canopy architecture on relationships between various vegetation indices and LAI and Fpar: A computer simulation. Remote Sensing Reviews 10, 309–347. https://doi.org/10.1080/02757259409532252

Haboudane, D., Miller, J.R., Pattey, E., Zarco-Tejada, P.J., Strachan, I.B., 2004. Hyperspectral vegetation indices and novel algorithms for predicting green LAI of crop canopies: Modeling and validation in the context of precision agriculture. Remote Sensing of Environment 90, 337–352. https://doi.org/10.1016/j.rse.2003.12.013

Haboudane, D., Miller, J.R., Tremblay, N., Zarco-Tejada, P.J., Dextraze, L., 2002. Integrated narrow-band vegetation indices for prediction of crop chlorophyll content for application to precision agriculture. Remote Sensing of Environment 81, 416–426. https://doi.org/10.1016/S0034-4257(02)00018-4

Huete, A., Justice, C., Liu, H., 1994. Development of vegetation and soil indices for MODIS-EOS. Remote Sensing of Environment 49, 224–234. https://doi.org/10.1016/0034-4257(94)90018-3

Qi, J., Chehbouni, A., Huete, A.R., Kerr, Y.H., Sorooshian, S., 1994. A modified soil adjusted vegetation index. Remote Sensing of Environment 48, 119–126. https://doi.org/10.1016/0034-4257(94)90134-1

Roujean, J.-L., Breon, F.-M., 1995. Estimating PAR absorbed by vegetation from bidirectional reflectance measurements. Remote Sensing of Environment 51, 375–384. https://doi.org/10.1016/0034-4257(94)00114-3

Rouse, J.W., Haas, R.H., Schell, J.A., Deering, D.W., others, 1974. Monitoring vegetation systems in the Great Plains with ERTS. NASA Spec. Publ 351, 309.

Sims, D.A., Gamon, J.A., 2002. Relationships between leaf pigment content and spectral reflectance across a wide range of species, leaf structures and developmental stages. Remote Sensing of Environment 81, 337–354. https://doi.org/10.1016/S0034-4257(02)00010-X
